# Asymmetric interaction and indeterminate fitness correlation between cooperative partners in the fig–fig wasp mutualism

**DOI:** 10.1098/rsif.2011.0063

**Published:** 2011-04-13

**Authors:** Rui-Wu Wang, Bao-Fa Sun, Qi Zheng, Lei Shi, Lixing Zhu

**Affiliations:** 1 State Key Laboratory of Genetic Resources and Evolution; Ecology, Conservation, and Environment Center, Kunming Institute of Zoology, Chinese Academy of Science, Kunming, Yunnan 650223, Republic of China; 2 Statistics and Mathematics College, Yunnan University of Finance and Economics, Kunming, Yunnan 650221, Republic of China; 3 Department of Mathematics, Hong Kong Baptist University, Hong Kong 999077, Republic of China

**Keywords:** asymmetric cooperation, tragedy of the commons, mutualism, chaotic oscillation, fig–fig wasp, interference competition

## Abstract

Empirical observations have shown that cooperative partners can compete for common resources, but what factors determine whether partners cooperate or compete remain unclear. Using the reciprocal fig–fig wasp mutualism, we show that nonlinear amplification of interference competition between fig wasps—which limits the fig wasps' ability to use a common resource (i.e. female flowers)—keeps the common resource unsaturated, making cooperation locally stable. When interference competition was manually prevented, the fitness correlation between figs and fig wasps went from positive to negative. This indicates that genetic relatedness or reciprocal exchange between cooperative players, which could create spatial heterogeneity or self-restraint, was not sufficient to maintain stable cooperation. Moreover, our analysis of field-collected data shows that the fitness correlation between cooperative partners varies stochastically, and that the mainly positive fitness correlation observed during the warm season shifts to a negative correlation during the cold season owing to an increase in the initial oviposition efficiency of each fig wasp. This implies that the discriminative sanction of less-cooperative wasps (i.e. by decreasing the egg deposition efficiency per fig wasp) but reward to cooperative wasps by fig, a control of the initial value, will facilitate a stable mutualism. Our finding that asymmetric interaction leading to an indeterminate fitness interaction between symbiont (i.e. cooperative actors) and host (i.e. recipient) has the potential to explain why conflict has been empirically observed in both well-documented intraspecific and interspecific cooperation systems.

## 1. Introduction

Explaining the evolution of cooperation and how cooperative systems achieve stability remains one of the most debated problems in both the biological and the social science communities [1,2]. The empirical observations and analysis have shown that cooperative actors, in fact, take a mixed strategy of either cooperating with the recipient or cheating/competing with the recipient in both intraspecific (e.g. [3–5]) and interspecific cooperative systems (e.g. [6–9]). One of the most interesting questions is how cooperation can be stably maintained as conflict increases between the recipient and the cooperative actors under the number increase of involved partners or the further action repeat of the cooperative actors in systems with a limited common resource, and the increased conflict might lead to breakdown of the cooperative interaction, even when there is a strong genetic relatedness or reciprocal exchange between partners [10–13]. Such a breakdown in cooperative behaviour represents a ‘tragedy of the commons’ in the evolution of cooperation [14].

Classical theories of evolution of cooperation based on an assumption of symmetrical interactions argued that cooperation could be maintained either through the self-restraint of cooperative actors or through spatial heterogeneity created by the recipient because of high genetic similarity or reciprocal exchange between the cooperative partners [10,15–18]. However, theoretical explorations showed that taking a non-cooperative strategy or even being a parasite within these systems will be more advantageous than the cooperating strategy of self-restraint (i.e. not competing with partners); after the utilization of the common resource is saturated, individuals employing the pure cooperation strategy (not competing with others) may not receive any fitness benefit [10,19,20]. The spatial heterogeneity created by the recipient is also not a credible mechanism for maintaining the stability of cooperative systems. Recent theoretical advances have shown that heterogeneity will often emerge but fails to promote the evolution of cooperation, because the mutant that can overcome the spatial heterogeneity to use the common resource at the expense of recipient will have higher fitness than cooperating actors in a limited local space [21].

Empirical observation and data analysis have further shown that self-restraint of cooperative actors or spatial heterogeneity created by recipients cannot sufficiently maintain the stable cooperative interaction in both intraspecific and interspecific cooperation systems. In almost all of the well-documented intraspecific cooperation systems, the literature shows that cooperative actors (i.e. the subordinates) are in fact competing with the recipient (i.e. king or queen) for the common resource or tend to be lazy to reproduce by themselves (e.g. in lion, see [3]; in mole rodent, see [5]; in bee and ant, reviewed by [22]). Explorations of interspecific cooperation systems also show that the ‘honest’ cooperative actors of a mutualism (i.e. the symbiont) can compete with their host (i.e. cooperative recipient) in several well-known mutualism systems (e.g. in fig–fig wasp mutualism, see [6,23,24]; in mutualism between moth *Greya politella* and plant *Lithophragma parviflorum*, see [25]). Our previous manipulative experiments with the fig–fig wasp mutualism have shown that the cooperative interaction between the fig and its pollinator wasps will transform into a conflict interaction when the unused common resource (unused female flowers of figs) decreases [9].

That both cooperation and conflict exist in these cooperative systems show that pure cooperation equilibrium might not exist in cooperative systems, and that the systems that display cooperation might be in a non-equilibrium state (e.g. a chaotic system, see [26–28]). In non-equilibrium systems, the behavioural impact of one partner on the other partner is indeterminate, and therefore the interaction between involved partners cannot be expected to be constant. However, such systems with indeterminate behavioural interactions between particles/elements/partners could be maintained at a steady state through asymmetric interaction (e.g. [29] nuclear physics; see [30] thermodynamics; see [31] economics). Theoretically, the control of initial perturbation can favour the steady state in non-equilibrium systems [32,33]. In cooperative systems, asymmetric interaction between the cooperative actors and recipients (e.g. density-dependent interference competition among cooperative actors, but not among recipients; the recipient might discriminatively sanction against non-cooperative actors but reward the cooperative actors) might lead to a non-equilibrium state, but the control of initial common resource utilization efficiency will favour the steady-state maintenance of the cooperation systems (see mathematical simulation of [20]).

In this paper, we use direct fitness correlation analysis to examine whether the well-documented interspecific mutualism between figs and fig wasps is stabilized by an asymmetric interaction. In the fig–fig wasp mutualism, figs provide pollinator wasps with part of their female flowers for oviposition and the eggs of pollinators develop into adults in the ovaries of flowers (galls); in exchange, wasp pollinators carry pollen from the mature fruits to receptive fruits. In the process of oviposition, pollen is dispersed to female flowers by foundresses (pollinators in fruit cavities), and part of fertilized female flowers develops viable seeds. In monoecious figs, because both pollinator offspring and viable seeds of figs use the same resource (i.e. female flowers), there should be conflict in using female flowers, which could disrupt the cooperation system [6,9,23].

It is generally believed that spatial heterogeneity (i.e. the structural barrier of flowers) created by host plants and/or an evolutionary constraint on fig wasps prevent pollinators (symbionts) from over-using common resource at the expense of host plants, thus keeping mutualistic cooperation in a stable equilibrium state. However, further quantitative examination has shown that neither the hypothesized spatial heterogeneity (structural barrier of flowers such as the heterogeneity in style or pedicel length) created by plants nor the evolutionary constraint of symbionts in the fig–fig wasp mutualism to be sufficient to prevent fig wasps from over-using female flowers at the expense of viable seeds [9,24,34,35]. This is in spite of many suggested mechanisms of spatial heterogeneity or evolutionary constraint for symbionts.

In a cooperative system, if spatial heterogeneity or self-restraint can prevent a direct conflict between recipient and cooperative actors, only a positive fitness correlation (i.e. viable seed–wasp offspring correlation) can be observed and a negative fitness correlation (conflict) will not be present even when the use of the common resource is saturated [9,10,17]. However, positive fitness correlation (i.e. a cooperative interaction) can also be observed if the common resource is unsaturated, given that genetic relatedness or reciprocal exchange between the recipient and cooperative actors is higher than the cost/benefit ratio [11,12]. In the research described in this paper, we used fig (*Ficus racemosa*) and its obligate pollinator *Ceratosolen fuscicep*s to examine how the steady state in a non-equilibrium state between figs and their pollinator wasps is maintained under the variation of environmental or ecological factors through interference competition among foundresses (i.e. pollinator wasps in fruit cavities). The interference competition among the foundresses that could decrease the oviposition of the flowers [36], however, might also decrease the pollination of the flowers. Following from our previous paper [36], in this paper we would like to explore the remaining problem on why both cooperative (i.e. positive fitness correlation) and conflict interactions (i.e. negative fitness correlation) can be observed between figs and their pollinator wasps under the interference competition among the fig wasps.

## 2. Material and methods

### 2.1. Study species

*Ficus racemosa* Linn. (*Ficus sycomorus*) is distributed from India to Australia. *Ficus racemosa* is a large tree that can reach 30 m high and bears cauliflorous fruits synchronously within the tree in very large numbers. It grows mainly in moist valleys or along rivers. Trees of *F. racemosa* usually grow in groups of five to 10. *Ficus racemosa* is pollinated by the wasp species *C. fusciceps* Mayr (Agaonidae). The foundress number per receptive fruit of *F. racemosa* usually range from 1 to 30, but can sometimes reach more than 70. The averaged foundress number per receptive syconium varies greatly with different habitat sites and seasons [37].

### 2.2. Introduction method

Interference competition, which should exist if foundresses (cooperative actors) simultaneously enter fruit cavities, can be excluded partly or totally if foundresses sequentially enter fruit cavities at time intervals [36,38]. We designed an experiment to examine whether there was interference competition among pollinator fig wasps, and to observe whether there was a negative fitness correlation when interference competition was excluded. In one set of treatments, we simultaneously introduced pollinators (manually collected wasps) into receptive fruits within 30 min. In these experiments of simultaneous introduction of foundresses, we conducted six treatments in which two, five, seven, nine, 15 and 20 foundresses in each treatment were simultaneously introduced into each receptive syconium.

With sequential introduction of foundresses, we conducted four treatments in which two, five, seven and nine foundresses in each treatment were separately introduced. In the two-foundress treatment, we introduced one on the first day at 09.00 h, and the second on the second day at 09.00 h for each receptive syconium. In the other three treatments with, respectively, five, seven and nine foundresses in total for each receptive syconium, we introduced two on the first day at 09.00 h and then sequentially introduced two, or one if only one was left, every 3–4 h in the day time or 10–12 h in the evening. Foundresses were collected using an insect net from the air surrounding receptive syconia.

In the above comparison experiments between the simultaneous and sequential introductions, the data of oviposition of flowers with two, five, seven and nine foundresses have appeared in our previous paper [36]. Because the interference competition occurs in both oviposition and pollination of the foundresses, in this paper we would like to see whether there is a difference in the intensity of interference competition among the foundresses between the oviposition and pollination of the flowers.

Because the oviposition efficiency of each foundress (i.e. initial value of oviposition) differs between morning and afternoon (table 1), we conducted an extra four treatments with both sequential and simultaneous introduction with nine foundresses to compare whether the oviposition efficiency of each foundress affects final fitness correlation in the presence of density-dependent interference competition among the foundresses. In these four extra experiments, the first introduction was at 06.00 h and at 15.00 h, while the others were treated in the manner identical to the other introductions. These extra four treatments were only conducted in one sample site (crop field) with the same two trees to ensure that other environmental conditions were the same.

**Table 1.** Factors that might influence oviposition efficiency of foundresses (oviposition efficiency: galled flowers per foundress), (mean ± s.e.). The experimental data showed that the lifespan of the pollinator was strongly affected by the temperature and humidity of environmental conditions [24,39], which might result in the oviposition efficiency difference between morning and afternoon, or between warm and cold season. The data in this table also show that the fruit diameter will significantly affect the oviposition efficiency of each foundress, conforming to the correlation analysis result in §3. Here, O/F is the offspring (gall) number per foundress, and FD is the fruit diameter (millimetre).

factors	between different fruit diameters (nine foundresses)	between different fruit diameters (two foundresses)	between morning and afternoon (two foundresses)	between warm and cold seasons (two foundresses)
treatment 1	O/F: 114.1 ± 5.4 (*n* = 20)	O/F: 264.5 ± 11.2 (*n* = 21)	O/F: 502.3±13.6 (09.00 h, *n* = 20)	O/F: 381.8 ± 30.4 (*n* = 34; Apr., Mar., Sep.)
	FD: 41.2 ± 1.1	FD: 45.8 ± 0.6		
treatment 2	O/F: 155.9 ± 12.2 (*n* = 20)	O/F: 273.4 ± 9.0 (*n* = 20)	O/F: 229.9 ± 9.0 (15.00 h, *n* = 20)	O/F: 812.1 ± 56.9 (*n* = 34; Nov., Dec.)
	FD: 50.3 ± 1.0	FD: 36.4 ± 0.2		
comparison between two treatments	O/F: *t* = 3.1; d.f. = 38, *p* < 0.01; FD: *t* = 6.3, d.f. = 38, *p* < 0.001	O/F: *t* = 0.6; d.f. = 39, *p* > 0.05; FD: *t* = 14.9, d.f. = 39, *p* < 0.001	*t* = 16.7, d.f. = 38, *p* < 0.001	*t* = 6.7, d.f. = 66; *p* < 0.001

In the experiment designed to determine oviposition efficiency, we manually introduced two foundresses into receptive fruits. Syconia pollinated by a single foundress often abort [40], and there is limited interference competition for pollination or oviposition between foundresses in large figs (e.g. those of *F. racemosa*) when foundress number is two [38]. The mean pollination and oviposition per foundress for syconia-containing two foundresses can therefore be used to evaluate oviposition efficiency of foundresses. We conducted such experiments in both the warm and cold seasons, and morning and afternoon within the same day.

In these experiments, all of the treated fig fruits were enclosed in nylon bags during development to prevent any oviposition by other wasps. In the above experiments, we selected two trees in a crop field and another two trees in a locally fragmented forest.

### 2.3. Natural data collection

We also counted the number of viable seeds in each fig, the number of fig wasp offspring (galled flower number) and the amount of unused common resource (vacant female flowers) over years from four sampling sites at the centre of the primary forest, edge of the primary forest, within a locally fragmented forest and from isolated fig trees by rubber trees, roads or crop fields. We collected these data over most months of the year. Oviposited flowers (galls), viable seeds and vacant female flowers were counted in premature syconia (pre-D phase) or mature syconia (D phase). We vertically cut each syconium into eight slices passing through the ostiole, and then randomly selected two or three slices to count all galls, seeds and vacant flowers. Owing to the difficulty of counting all vacant flowers, only galls and seeds of the remaining five or six slices were counted. We then calculated the percentage of the developed flowers (galls + seeds) per syconium and estimated the total number of the female flowers per syconium using the following calculation: the total number of the female flowers = (total galls + total seeds)/proportion of the developed flowers.

### 2.4. Correlation analysis

Cooperation or conflict interaction can essentially be described by the direct or inclusive fitness correlation coefficient between involved players in both intraspecies and interspecies cooperation systems [1,9,20,41,42]. However, if the cooperation interaction of involved players is not in a stable equilibrium (e.g. chaotic oscillation) a constant fitness relationship between involved players cannot be expected, possibly presenting an indeterminate interaction between the individual players. On the other hand, the whole system could still be in a stable state (e.g. [32,33,43]). In the case where there is no constant relationship between interacting partners, the fitness regression coefficient using simple linear regression or any other parametric correlation analysis will be the averaged value of their oscillating correlation coefficients, and therefore would not describe the true relationship between the involved players [9,43]. In an indeterminate system (e.g. chaos system), the direct fitness or inclusive fitness correlation coefficient (i.e. fitness regression coefficient) between recipient and cooperative actors should vary or oscillate with variation of affecting factors, which could be the frequency of cooperative actors, or common resource availability or other factors [9,20,27,43].

In the manipulated experiments, because the effect of indeterminate environmental or ecological factors was mostly prevented, we used a simple linear regression to analyse the correlation between the viable seeds and egg deposition of the foundresses (i.e. galls), when the foundresses were simultaneously introduced into the syconium cavity. When the foundresses were sequentially introduced into the syconium cavity, we used quadratic function to fit the distribution pattern of the viable seeds as a function of wasp offspring. This analysis was able to determine whether any positive contribution of egg deposition translated to a negative effect on seed production.

Because the correlation coefficient between the viable seeds and wasp offspring number varies so greatly a constant correlation coefficient cannot be expected under the variation of the environmental change, even in the experiments given the foundress number (table 2). Under natural conditions, the factors that will affect the fitness correlation between fig and fig wasps will be indeterminate, and therefore the linear regression or generalized linear regression which assumes that the function response between the interacted variables could be given cannot be used to estimate such indeterminate interaction between fig and fig wasps. For the data collected from the natural condition, we used varying correlation coefficient with non-parametric estimation to describe the correlation between viable seeds of figs and wasp offspring of fig wasps as a function of other factors. Through such a method, we can describe how the fitness interaction between figs and fig wasps varied with changes in other factors.

**Table 2.** The correlation coefficient between viable seeds and the galled female flowers in different conditions when the foundress number is 9, controlling for the syconium size and the total female flower number. The results here showed, given the foundress number, that the correlation coefficient between viable seeds and galled female flowers varies greatly under different introduction treatments, as well as different environmental conditions.

sample site and introduction time	sample size	introduction treatment	total female flowers per syconium (Mean ± s.e.)	syconium size (Mean ± s.e.)	correlation coefficient
samples from different habitat sites and different introduction treatments					
crop field (at 09.00 h)	20	simultaneous	5443.1 ± 161.8	50.3 ± 1.0	0.74***
crop field (at 21.00 h)	21	sequential	5815.9 ± 295.8	50.4 ± 0.8	−0.67***
fragmented forest (at 21.00 h)	20	simultaneous	4084.6 ± 81.1	41.2 ± 1.1	0.68**
fragmented forest (at 21.00 h)	20	sequential	4266.1 ± 82.7	38.6 ± 0.2	−0.07 n.s.
samples from the same habitat site with different treatments					
crop field (at 06.00 h)	20	simultaneous	4886.2 ± 96.9	46.1 ± 0.3	0.06 n.s.
crop field (at 06.00 h)	20	sequential	4791.8 ± 67.3	46.0 ± 0.3	−0.96***
crop field (at 15.00 h)	20	simultaneous	4701.5 ± 98.0	45.5 ± 0.3	0.75**
crop field (at 15.00 h)	20	sequential	4966.8 ± 91.0	46.9 ± 0.4	−0.64**

n.s. is not significant at *p* = 0.05.

** *p* < 0.01.

*** *p* < 0.001.

The varying coefficient method with non-parametric estimation does not pre-specify functional forms of the involved variables, but uses information purely from data to estimate a curve/function. Such varying coefficient method is obviously more flexible and robust to fit data than any parametric method, because if a parametric structure of the model is pre-assumed, there is a risk of the wrong model and conclusions [44,45]. When the functional response between the interacted variables can be given to be a specific form, the varying coefficient is then reduced to the traditional parametric correlation analyses, such as linear regression or generalized linear regression methods. As such, the varying coefficient is more general and more flexible for use in empirical data analyses, and then, different from the traditional methods, it might help us identify whether or not species interaction varies with respect to other factors, and what factors will be crucial to maintain or change the species interaction. For details of the varying coefficient analysis model and the comparison between the varying coefficient method and traditional linear regression or generalized linear regression see the electronic supplementary material, online appendix or the paper by Shi *et al*. [46].

## 3. Analyses and results

**Figure 1.**
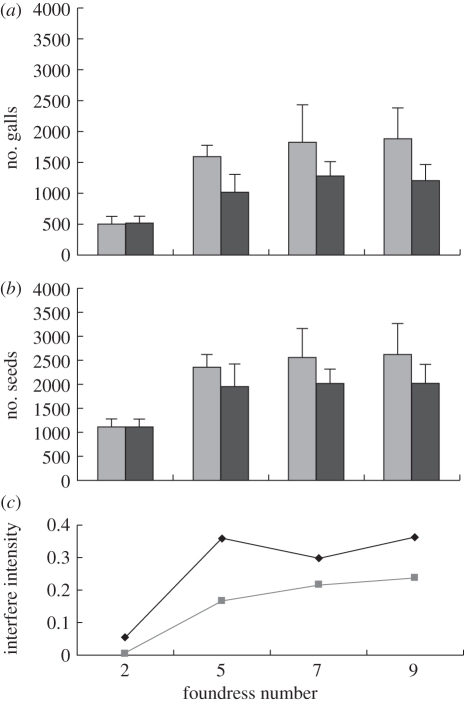
The effect of foundress abundance on the intensity of interference competition between the foundresses on the gall and seed production in *F. racemosa*. (*a*) Gall production (mean ± s.d.), which is similar to fig. 5 of Wang *et al*. [36], (*b*) viable seed production (mean ± s.d.), (*c*) interference competition intensity ((number of sequential introduction—number of simultaneous introduction)/number of sequential introduction). That the efficiency of pollination per foundress is higher than oviposition (i.e. initial value) is selected by the discriminative sanction of less cooperative or cheating wasps but reward to the cooperative wasps by host ([40]; R.-W. Wang *et al*. 2009, unpublished data). (*a*,*b*) Light grey bars, foundress sequential entry; dark grey bars, foundress simultaneous entry. (*c*) Diamonds with solid line, galls; squares with solid line, seeds.

Similar to the results of Bronstein *et al*. [38], there was no interference competition when there were two foundresses, and both viable seed production and wasp offspring (gall) production were not significantly different between treatments of simultaneous and sequential entry of fig wasps (figure 1*a,b*). In contrast, there was lower oviposition and pollination with simultaneous entry of pollinators in treatments with five, seven and nine foundresses than with sequential entry. The mean comparison with general linear model including the syconium size and the total number of female flowers as covariates for seed production were for five foundresses, *F*_1,78_ = 11.116, *p* < 0.001; for seven foundresses, *F*_1,79_ = 37.912, *p* < 0.001; and for nine, *F*_1,79_ = 47.979, *p* < 0.001. For gall production, results were for five foundresses, *F*_1,78_ = 44.332, *p* < 0.001; for seven foundresses, *F*_1,79_ = 66.369, *p* < 0.001; and for nine, *F*_1,79_ = 67.910, *p* < 0.001. The intensity of interference competition on the production of galled flowers is higher than on the viable seeds (figure 1*c*).

**Figure 2.**
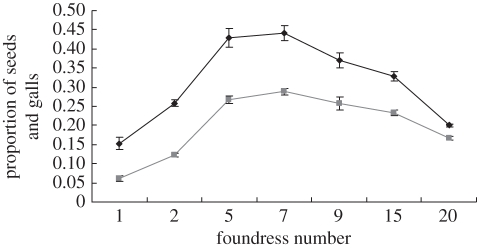
The production of wasp offspring and viable seeds as a function of the number of simultaneously entering foundresses (mean ± s.e.). The data were collected from two trees in a crop field and sample size of each treatment greater than 20. Diamonds with solid line, seeds; squares with solid line, galls.

**Figure 3.**
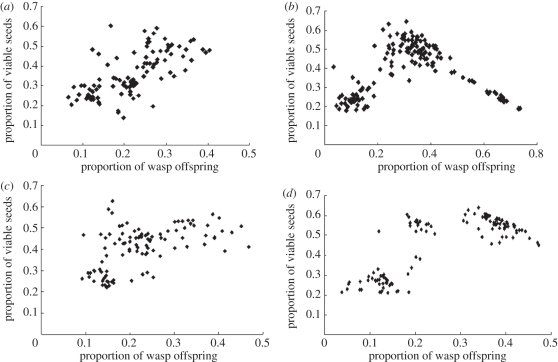
Proportion of seeds (seed number/total female flower number) as a function of the proportion of wasp offspring (galled flower number/total female flower number) in *F. racemosa* with different treatments. (*a*,*b*) Collected from crop field. (*a*) Foundresses introduced simultaneously; the foundress numbers were two, three, five, seven and nine with sample size 21, 20, 20, 20 and 20, respectively (linear: *n* = 101, *r* = 0.67, *p* < 0.001). (*b*) Foundresses sequentially introduced; foundress numbers were one, two, five, seven and nine with sample size 21, 20, 20, 23 and 65, respectively (quadratic: *F* test = 201.87, *p* < 0.001). (*c*,*d*) Collected from locally fragmented forest. (*c*) Foundresses introduced simultaneously; foundress numbers were two, three, five, seven and nine with sample size 20 for each treatment (linear: *n* = 100, *r* = 0.52, *p* < 0.001). (*d*) Foundresses sequentially introduced; foundress numbers were one, two, three, five, seven and nine with sample size 20 samples for each treatment (quadratic: *F* test = 23.14, *p* < 0.001).

**Figure 4.**
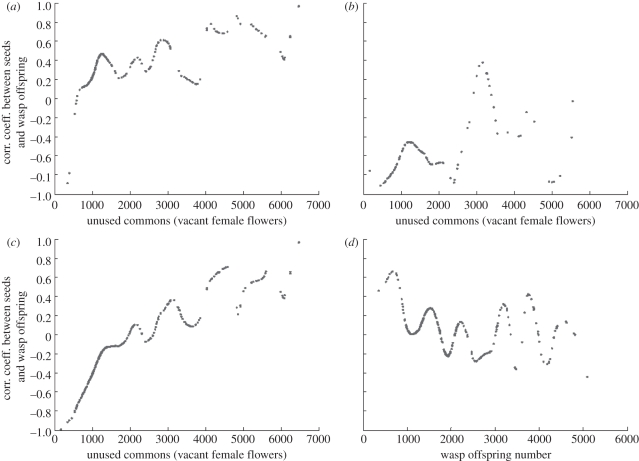
The Pearson correlation coefficient between the fitness of figs (viable seeds) and the fitness of pollinator wasps (offspring number) of cooperating species using a non-parametric estimation method. (*a*) Samples collected in the warm season (*n* = 218, March–October). (*b*) Samples collected in the cold season (*n* = 134, November–February). (*c*) Fitness correlation coefficient between seeds and wasp offspring as function of unused commons (vacant female flowers; *n* = 352, over years). (*d*) Fitness correlation coefficient between seeds and wasp offspring as function of wasp offspring (*n* = 352, over years). The bandwidth *h* is 400, which was obtained by cross-validation method.

With simultaneous entry of pollinators, the nonlinear amplification of interference competition among the foundresses cancelled out extra pollination and oviposition after the number of foundresses reached a threshold (figure 2). The interference competition among foundresses decreased the common resource utilization (i.e. developed female flowers) in both pollination and oviposition. Such a decreased utilization of common resource from interference competition among wasps makes the common resource utilization unsaturated. However, in experiments with sequential entry of pollinators, the viable seed production was obviously an inversed U-shape as a function of wasp offspring production, which differed from positive correlation for simultaneous entry (figure 3). The correlation coefficient between viable seeds and wasp offspring varies greatly in the experiments with the change of foundress numbers, introduction methods and habitat sites (tables 2 and 3). In the naturally collected data, the impact of wasp offspring on production of viable seeds (indicated by correlation coefficients) oscillated with the change in density of wasp offspring or the availability of unused common resource (vacant female flowers; figure 4).

**Table 3.** The correlation coefficient between viable seeds and the galled female flowers in different conditions and foundress number, controlling for the syconium size and the total female flower number. The results here showed that the correlation coefficient between viable seeds and galled female flowers varies greatly under different introduction treatments and different foundress numbers, as well as different environmental conditions.

sample site and introduction time	foundress number	sample size	introduction treatment	total female flowers per syconium (mean ± s.e.)	syconium size (mean ± s.e.)	correlation coefficient
crop field	2	21	simultaneous	4483.0 ± 104.5	45.8 ± 0.6	0.15 n.s.
crop field (at 21.00 h)	2	20	sequential	4360.7 ± 107.6	44.4 ± 0.5	0.26 n.s.
fragmented forest (at 21.00 h)	2	20	simultaneous	4136.5 ± 66.9	36.4 ± 0.2	−0.29 n.s.
fragmented forest (at 21.00 h)	2	20	sequential	4055.6 ± 45.2	35.9 ± 0.5	0.50 n.s.
crop field (at 09.00 h)	5	20	simultaneous	4698.7 ± 86.4	48.2 ± 1.1	0.29 n.s.
crop field (at 21.00 h)	5	20	sequential	4753.9 ± 61.1	49.5 ± 40.9	−0.03 n.s.
fragmented forest (at 21.00 h)	5	20	simultaneous	4169.3 ± 107.8	40.2 ± 0.6	0.07 n.s.
fragmented forest (at 21.00 h)	5	20	sequential	4217.8 ± 36.9	39.3 ± 0.8	−0.20 n.s.
crop field (at 09.00 h)	7	20	simultaneous	4393.8 ± 50.7	50.7 ± 0.5	0.40 n.s.
crop field (at 21.00 h)	7	23	sequential	5578.1 ± 260.8	52.1 ± 0.8	−0.401 n.s.
fragmented forest (at 21.00 h)	7	20	simultaneous	4075.1 ± 68.2	43.0 ± 0.6	−0.26 n.s.
fragmented forest (at 21.00 h)	7	20	sequential	4309.3 ± 29.1	36.0 ± 0.3	−0.79***

n.s. is not significant at *p* = 0.05.

*** *p* < 0.001.

The distribution patterns of viable seed production as a function of galled flower number differ between different sample sites (figure 3). This is expected because the interference competition impacts depend significantly on fruit diameters and the total number of female flowers, and these parameters differ between these two sample sites. In the simultaneous introduction experiment with nine foundresses, the correlation analysis showed that the fruit diameter was positively correlated with oviposition efficiency of foundresses (galled flower number per foundress), (*n* = 40, *r* = 0.47, *p* < 0.05). The oviposition efficiency of foundresses were also significantly correlated with the total number of female flowers (*n* = 40, *r* = 0.53, *p* < 0.001). The fruit diameter size and the total female flowers of samples from the crop field are on average larger than from the locally fragmented forest (diameter: *t* = 25.986, d.f. = 365, *p* < 0.001; female flowers: *t* = 11.175, d.f. = 365, *p* < 0.001).

In the fig–fig wasp mutualism, the oviposition efficiency per foundress varies greatly across different ecological or environmental conditions. In our experiments, the foundresses had higher oviposition efficiency in the morning than in the afternoon, and in particular demonstrated higher efficiency in the cold than in the warm season (table 1). This is because foundresses have much longer lifespans at lower temperature [24] and higher humidity [39]. In the morning, the humidity is higher than in the afternoon and the temperature is usually lower than in the afternoon. In table 2, the foundress number is the same in all of the treatments, but the fitness interaction between viable seeds and galled flowers varies greatly under different foundress introduction treatments (i.e. simultaneous or sequential introduction), in different sample sites with different fruit size or total female flower number, and under different initial values of female flower utilization by foundresses at different introduction times (i.e. morning or afternoon; table 1).

The pollination efficiency of foundresses is higher than oviposition efficiency of foundresses (figure 1), leading to faster rates of pollination saturation than oviposition saturation in the female flowers of figs. When female flower utilization is saturated (i.e. flowers are either pollinated or oviposited), an increase in egg deposition leads to a decrease in seed production, and therefore a negative fitness interaction could be observed. In the warm season, the fitness interaction between figs and fig wasps was mainly positively correlated, but mainly negative in the cold season (figure 4). Such seasonally dependent seed–wasp offspring interaction patterns result because the initial rate of egg deposition (i.e. egg deposition efficiency of each foundress) in the cold season is much higher than in the warm season (table 1).

## 4. Discussion

The results of our investigation of interspecific cooperation between figs and fig wasps show that a local cooperative equilibrium between recipient (e.g. hosted figs) and cooperative actors (e.g. pollinator wasps) can be maintained by asymmetric interference competition (i.e. interference competition was mainly among the cooperative actors, not directly for the recipient). The interference competition among the cooperative actors decreases the common resource utilization by cooperative actors and kept the common resource utilization unsaturated, thus maintaining the cooperative interaction (i.e. positive fitness interaction; figures 2 and 3). However, the impact of interference competition among the cooperative actors will be greatly affected by ecological or environmental constraints (e.g. dispersal barrier, number of cooperative actors, availability of common resource and common resource utilization efficiency of foundresses), and such ecological or environmental constraints might be indeterminate. The fitness interaction shown in figure 4 therefore becomes a stochastic process, and the probability of a positive fitness interaction differs under different ecological or environmental conditions.

Our results imply that the common resource of female flowers for recipient (host plant) and cooperative actors (symbionts) was global rather than local, and direct fitness conflict between fig plants and pollinator wasps would occur after the common resource utilization was saturated in some specific situations. For example, in the environment with higher humidity or lower temperature, the oviposition efficiency can be increased, or when the foundresses could enter syconium with a longer interval, the interference competition among the foundresses can be lessened. This observation suggests that genetic relatedness or reciprocal exchange may be important (by creating spatial heterogeneity or exercising recognition discrimination against additional cooperative actors or cheating individuals) but is not by itself a sufficient explanation for how cooperation is maintained. This is the first direct empirical evidence in support of the argument that the Nash equilibrium or evolutionary stable strategy in cooperative systems may be inaccessible [19,47] and that an explanation of cooperation based on spatial chaotic oscillations might be more credible [26,27]. The genetic relatedness or reciprocal exchange might just be a pivot rather than an aim of evolution towards cooperation [28], and the higher genetic relatedness or reciprocal exchange will reduce the probability of competition [20].

Genetic relatedness or reciprocity exchange results from limited dispersal from the original colony [17,48]. However, limited dispersal might also result in competition between involved partners, not only between cooperative actors and the recipient, but also among the cooperative actors (i.e. pollinator wasps in fig–fig wasp mutualism, see [36]) [12]. As is shown in figures 1 and 3, more intensive competition between the cooperative actors means there will be a lower probability of the common resource being over-exploited, and therefore a less probability of conflict in the interaction between cooperative actors and the recipient. Theoretically, higher dispersal difficulty for cooperative actors (creating higher genetic relatedness or reciprocity between the recipient and the cooperative actors) will increase the interference competition among the cooperative actors, and therefore higher rates of cooperation will be expected in systems with higher dispersal difficulty [20]. The higher dispersal difficulty for cooperative actors will make the sanction or repression of less-cooperative wasps by host or dominant individuals more credible [40]. Through such a control of initial value (e.g. by decreasing the egg deposition efficiency per fig wasp in fig–fig wasp mutualism), the steady state in non-equilibrium of cooperation systems might be maintained [32,33].

Essentially, the cooperative interaction is maintained by density-dependent interference competition (i.e. a process of nonlinear amplification) owing to suppression of resource consumption, which prevents resource use from being saturated (figures 3 and 4*c*). Variation in the availability of a common resource, the intensity of interference competition or common resource utilization efficiency will greatly affect the saturation of the common resource utilization, whereas the availability of unused common resource will directly determine the fitness correlation relationship between the symbiont and the host (figure 4*c*). Further, the above variables are also greatly affected by ecological or environmental factors. As such, the relationship between the symbiont and the host, therefore, might be indeterminate. Our discovery of the factors influencing this oscillating relationship reinforces previous findings that the interaction between symbionts and hosts might oscillate between mutualism and antagonism across temporal or spatial variability [24,25,41,49,50]. The interaction between the reciprocal mutualists might be conditional [51]. This also explains why increased density of cooperative actors leads to direct conflict (competition) between cooperative actors and recipient for a limited common resource [9,12].

Density-dependent (i.e. nonlinear) interference competition among cooperative actors might be a general mechanism for maintaining cooperation rather than a special mechanism of the fig–fig wasp mutualism. In intraspecific cooperative systems, there exist dispersal barriers for involved partners and a larger number of cooperative actors than recipients [1,3,17], a condition similar to that of the fig–fig wasp interspecific cooperation [9,48]. Thus, competition among the cooperative actors might be more intense than between recipient and cooperative actors, owing to asymmetric interaction between recipient and cooperative actors. Such density-dependent interference competition among cooperative actors will lead to an indeterminate fitness interaction between cooperative actors and recipient [20,52]. Different fitness interactions might lead to different evolutionary and behavioural strategies. This could explain why cooperative actors take mixed strategies of cooperation or competition, rather than pure cooperation with the recipient in genotype, phenotype or behaviour [3,7,9,53].

A remaining question is what dynamics drive the players involved to pay evolutionary costs to punish cheating or less-cooperative individuals with the potential to disrupt the cooperative interaction [12,40,54]. If a high probability of cooperative interaction can be maintained by environmental or ecological restraint through the mechanism of mutual interference among cooperative actors, the net interest of the recipient will be greater for cooperation than conflict over an entire life cycle. It would be advantageous to the recipient (i.e. host of mutualism or dominant of intraspecific cooperation systems) to pay costs to maintain a stable cooperative interaction by punishing less cooperative or cheating behaviours of cooperative actors [52,55,56].

## References

[1] Frank S. A. 1998 Foundations of social evolution. New Jersey, NJ: Princeton University

[2] Gardner A. West S. A. 2004 Cooperation and punishment, especially in humans. Am. Nat. 164, 753–764 10.1086/425623 (doi:10.1086/425623) 29641920

[3] Heinsohn R. Packer C. 1995 Complex cooperative strategies in group-territorial African lions. Science 269, 1260–1262 10.1126/science.7652573 (doi:10.1126/science.7652573) 7652573

[4] Ratnieks F. L. W. Wenseleers T. 2005 Policing insect societies. Science 307, 54–56 10.1126/science.1106934 (doi:10.1126/science.1106934) 15637260

[5] Reeve H. K. 1992 Queen activation of lazy workers in colonies of the Eusocial naked mole-rat. Nature 358, 147–149 10.1038/358147a0 (doi:10.1038/358147a0) 1614546

[6] Anstett M. C. Bronstein J. L. HossaertMcKey M. 1996 Resource allocation: a conflict in the fig/fig wasp mutualism? J. Evol. Biol. 9, 417–428 10.1046/j.1420-9101.1996.9040417.x (doi:10.1046/j.1420-9101.1996.9040417.x)

[7] Pellmyr O. Leebens-Mack J. 2000 Reversal of mutualism as a mechanism for adaptive radiation in yucca moths. Am. Nat. 156, S62–S76 10.1086/303416 (doi:10.1086/303416) 29592582

[8] Thompson J. N. 1982 Interaction and coevolution. New York, NY: Wiley and Sons

[9] Wang R. W. Shi L. Ai S. M. Zheng Q. 2008 Trade-off between reciprocal mutualists: local resource availability-oriented interaction in fig/fig wasp mutualism. J. Anim. Ecol. 77, 616–623 10.1111/j.1365-2656.2008.01359.x (doi:10.1111/j.1365-2656.2008.01359.x) 18266694

[10] Doebeli M. Knowlton N. 1998 The evolution of interspecific mutualisms. Proc. Natl Acad. Sci. USA 95, 8676–8680 10.1073/pnas.95.15.8676 (doi:10.1073/pnas.95.15.8676) 9671737 PMC21135

[11] Taylor P. D. 1992 Altruism in viscous populations–an inclusive fitness model. Evol. Ecol. 6, 352–356 10.1007/BF02270971 (doi:10.1007/BF02270971)

[12] West S. A. Pen I. Griffin A. S. 2002 Conflict and cooperation—cooperation and competition between relatives. Science 296, 72–75 10.1126/science.1065507 (doi:10.1126/science.1065507) 11935015

[13] Williams G. C. 1966 Adaptation and natural selection. Princeton, NJ: Princeton University Press

[14] Hardin G. 1968 The tragedy of the commons. Science 162, 1243–1248 10.1126/science.162.3859.1243 (doi:10.1126/science.162.3859.1243) 5699198

[15] Axelrod R. 1984 The evolution of cooperation. New York, NJ: Basic Books

[16] Hamilton W. D. 1964 The genetical evolution of social behaviour. Theor. Biol. 7, 1–16 10.1016/0022-5193(64)90038-4 (doi:10.1016/0022-5193(64)90038-4) 5875341

[17] Hamilton W. D. 1972 Altruism and related phenomena, mainly in social insects. Ann. Rev. Ecol. Syst., 3, 193–232 10.1146/annurev.es.03.110172.001205 (doi:10.1146/annurev.es.03.110172.001205)

[18] Maynard Smith J. 1982 Evolution and the theory of games. Cambridge, UK: Cambridge university press

[19] Boyd R. Lorberbaum J. P. 1987 No pure strategy is evolutionarily stable in the repeated Prisoners-Dilemma game. Nature 327, 58–59 10.1038/327058a0 (doi:10.1038/327058a0)

[20] Wang R. W. Shi L. 2010 The evolution of cooperation in asymmetric systems. Sci. China Life Sci. 53, 139–149 10.1007/s11427-010-0007-6 (doi:10.1007/s11427-010-0007-6) 20596966

[21] Hauert C. Doebeli M. 2004 Spatial structure often inhibits the evolution of cooperation in the snowdrift game. Nature 428, 643–646 10.1038/nature02360 (doi:10.1038/nature02360) 15074318

[22] Ratnieks F. L. W. Wenseleers T. 2008 Altruism in insect societies and beyond: voluntary or enforced? Trends Ecol. Evol. 23, 45–52 10.1016/j.tree.2007.09.013 (doi:10.1016/j.tree.2007.09.013) 18082910

[23] Herre E. A. West S. A. 1997 Conflict of interest in a mutualism: documenting the elusive fig wasp seed trade-off. Proc. R. Soc. Lond. B 264, 1501–1507 10.1098/rspb.1997.0208 (doi:10.1098/rspb.1997.0208)

[24] Wang R.-W. Yang J.-X. Yang D.-R. 2005 Seasonal changes in the trade-off among fig-supported wasps and viable seeds in figs and their evolutionary implications. J. Integr. Plant Biol. 47, 144–152 10.1111/j.1744-7909.2005.00034.x (doi:10.1111/j.1744-7909.2005.00034.x)

[25] Thompson J. N. Fernandez C. C. 2006 Temporal dynamics of antagonism and mutualism in a geographically variable plant-insect interaction. Ecology 87, 103–112 10.1890/05-0123 (doi:10.1890/05-0123) 16634301

[26] Brembs B. 1996 Chaos, cheating and cooperation: potential solutions to the Prisoner's Dilemma. Oikos 76, 14–24 10.2307/3545744 (doi:10.2307/3545744)

[27] Nowak M. A. May R. M. 1992 Evolutionary games and spatial chaos. Nature 359, 826–829 10.1038/359826a0 (doi:10.1038/359826a0)

[28] Nowak M. A. Sigmund K. 1992 Tit-for-tat in heterogeneous populations. Nature 355, 250–253 10.1038/355250a0 (doi:10.1038/355250a0)

[29] Lee T. D. Yang C. N. 1956 Question of parity conservation in weak interactions. Phys. Rev. 104, 254–258 10.1103/PhysRev.104.254 (doi:10.1103/PhysRev.104.254)

[30] Prigogine I. 1969 Structure, dissipation and life; in Theoretical physics and biology (ed. Marois M.), pp. 23–52 Amsterdam, The Netherlands: North-Holland

[31] Stiglitz J. Walsh C. E. 2002 Economics, 3rd edn. New York, NY: W.W. Norton & Company press

[32] Hazledine S. Sun J. Wysham D. Downie J. A. Oldroyd G. E. D. Morris R. J. Costa M. 2009 Nonlinear time series analysis of nodulation factor induced calcium oscillations: evidence for deterministic chaos? PLoS ONE 4, e6637 10.1371/journal.pone.0006637 (doi:10.1371/journal.pone.0006637) 19675679 PMC2722092

[33] Shinbrot T. Grebogi C. Ott E. Yorke J. A. 1993 Using small perturbations to control chaos. Nature 363, 411–417 10.1038/363411a0 (doi:10.1038/363411a0)

[34] Nefdt R. J. C. Compton S. G. 1996 Regulation of seed and pollinator production in the fig fig wasp mutualism. J. Anim. Ecol. 65, 170–182 10.2307/5720 (doi:10.2307/5720)

[35] Yu D. W. Ridley J. Jousselin E. Herre E. A. Compton S. G. Cook J. M. Moore J. C. Weiblen G. D. 2004 Oviposition strategies, host coercion and the stable exploitation of figs by wasps. Proc. R. Soc. Lond. B 271, 1185–1195 10.1098/rspb.2003.2630 (doi:10.1098/rspb.2003.2630) PMC1691705 15306369

[36] Wang R.-W. Ridley J. Sun B.-F. Zheng Q. Shi L. Dunn D. W. Cook J. Zhang Y.-P. Yu D. W. 2009 Interference competition and high temperatures reduce the virulence of fig wasps and stabilize a fig wasp mutualism. PLoS ONE 4, e7802 10.1371/journal.pone.0007802 (doi:10.1371/journal.pone.0007802) 19915668 PMC2771911

[37] Wang R.-W. Yang C.-Y. Zhao G.-F. Yang J.-X. 2005 Fragmentation effects on diversity of wasp community and its impact on fig/fig wasp interaction in *Ficus racemosal*. J. Integr. Plant Biol. 47, 20–26 10.1111/j.1744-7909.2005.00003.x (doi:10.1111/j.1744-7909.2005.00003.x)

[38] Bronstein J. L. Vernet D. Hossaert-McKey M. 1998 Do fig wasps interfere with each other during oviposition? Entomol. Exp. Appl. 87, 321–324 10.1046/j.1570-7458.1998.00337.x (doi:10.1046/j.1570-7458.1998.00337.x)

[39] Dunn D. W. Yu D. W. Ridley J. Cook J. M. 2008 Longevity, early emergence and body size in a pollinating fig wasp—implications for stability in a fig-pollinator mutualism. J. Anim. Ecol. 77, 927–935 10.1111/j.1365-2656.2008.01416.x (doi:10.1111/j.1365-2656.2008.01416.x) 18624736

[40] Wang R.-W. Sun B.-F. Zheng Q. 2010 Diffusive co-evolution and mutualism maintenance mechanisms in a fig–fig wasp system. Ecology 91, 1308–1316 10.1890/09-1446.1 (doi:10.1890/09-1446.1) 20503864

[41] Piculell B. J. Hoeksema J. D. Thompson J. N. 2008 Interactions of biotic and abiotic environmental factors in an ectomycorrhizal symbiosis, and the potential for selection mosaics. BMC Biol. 6, 23 10.1186/1741-7007-6-23 (doi:10.1186/1741-7007-6-23) 18507825 PMC2430191

[42] Queller D. C. 1992 A general-model for Kin selection. Evolution 46, 376–380 10.2307/2409858 (doi:10.2307/2409858) 28564031

[43] Sugihara G. May R. M. 1990 Nonlinear forecasting as a way of distinguishing chaos from measurement error in time-series. Nature 344, 734–741 10.1038/344734a0 (doi:10.1038/344734a0) 2330029

[44] Hastie T. Tibshirani R. 1993 Varying-coefficient models (with discussion). J. R. Stat. Soc. 55, 757–796

[45] Zhu L. X. 2005 Nonparametric Monte Carlo Tests and their applications. New York, NY: Springer

[46] Shi L. Wang R. W. Zhu L. X. Zen W. M. Xu W. L. Zheng Q. In press *Varying coefficient analysis for indeterminate species interaction with non-parametric estimation, exemplifying with a fig-fig wasp system.* Chinese Sci. Bull

[47] Nowak A. M. 1990 An evolutionary stable strategy may be inaccessible. J. Theor. Biol. 142, 237–241 10.1016/S0022-5193(05)80224-3 (doi:10.1016/S0022-5193(05)80224-3) 2352434

[48] Frank S. A. 1994 Genetics of mutualism: the evolution of Altruism between Species. J. Theor. Biol. 170, 393–400 10.1006/jtbi.1994.1200 (doi:10.1006/jtbi.1994.1200) 7996864

[49] Gomulkiewicz R. Nuismer S. L. Thompson J. N. 2003 Coevolution in variable mutualisms. Am. Nat. 162, S80–S93 10.1086/378705 (doi:10.1086/378705) 14583859

[50] Morris W. F. Wilson W. G. Bronstein J. L. Ness J. H. 2005 Environmental forcing and the competitive dynamics of a guild of cactus-tending ant mutualists. Ecology 86, 3190–3199 10.1890/05-0465 (doi:10.1890/05-0465)

[51] Bronstein J. L. 1994 Conditional outcomes in mutualistic interactions. Trends Ecol. Evol. 9, 214–217 10.1016/0169-5347(94)90246-1 (doi:10.1016/0169-5347(94)90246-1) 21236825

[52] Wang R. W. He J. Z. Wang Y. Q. Shi L. Li Y. T. 2010 Asymmetric interaction will facilitate the evolution of cooperation. Sci China Life Sci. 53, 1041–1046(doi:10.1007/s11427-010-4016-2) 20821304 10.1007/s11427-010-4016-2

[53] Griffin A. S. West S. A. 2002 Kin selection: fact and fiction. Trends Ecol. Evol. 17, 15–21 10.1016/S0169-5347(01)02355-2 (doi:10.1016/S0169-5347(01)02355-2)

[54] Bronstein J. L. 2001 The costs of mutualism. Am. Zool. 41, 825–839 10.1668/0003-1569(2001)041[0825:TCOM]2.0.CO;2 (doi:10.1668/0003-1569(2001)041[0825:TCOM]2.0.CO;2)

[55] Frank S. A. 1996 Policing and group cohesion when resources vary. Anim. Behav. 52, 1163–1169 10.1006/anbe.1996.0263 (doi:10.1006/anbe.1996.0263)

[56] Pellmyr O. Huth C. J. 1994 Evolutionary stability of mutualism between Yuccas and Yucca moths. Nature 372, 257–260 10.1038/372257a0 (doi:10.1038/372257a0)

